# Identification of factors that support successful implementation of care bundles in the acute medical setting: a qualitative study

**DOI:** 10.1186/s12913-017-2070-1

**Published:** 2017-02-07

**Authors:** Stuart A. Green, Derek Bell, Nicholas Mays

**Affiliations:** 1 Department of Health Services Research and Policy, London School of Hygiene and Tropical Medicine, 15-17 Tavistock Place, London, W1H 9SH UK; 2 NIHR CLAHRC Northwest London, Imperial College London, Chelsea and Westminster Hospital, Fulham Road, London, SW10 9NH UK

**Keywords:** Practice guidelines, Qualitative research, Delivery of health care, Evidence-based practice

## Abstract

**Background:**

Clinical guidelines offer an accessible synthesis of the best evidence of effectiveness of interventions, providing recommendations and standards for clinical practice. Many guidelines are relevant to the diagnosis and management of the acutely unwell patient during the first 24–48 h of admission. Care bundles are comprised of a small number of evidence-based interventions that when implemented together aim to achieve better outcomes than when implemented individually. Care bundles that are explicitly developed from guidelines to provide a set of related evidence-based actions have been shown to improve the care of many conditions in emergency, acute and critical care settings. This study aimed to review the implementation of two distinct care bundles in the acute medical setting and identify the factors that supported successful implementation.

**Methods:**

Two initiatives that had used a systematic approach to quality improvement to successfully implement care bundles within the acute medical setting were selected as case studies. Contemporaneous data generated during the initiatives included the review reports, review minutes and audio recordings of the review meetings at different time points. Data were subject to deductive analysis using three domains of the Consolidated Framework for Implementation Research to identify factors that were important in the implementation of the care bundles.

**Results:**

Several factors were identified that directly influenced the implementation of the care bundles. Firstly, the availability of resources to support initiatives, which included training to develop quality improvement skills within the team and building capacity within the organisation more generally. Secondly, the perceived sustainability of changes by stakeholders influenced the embedding new care processes into existing clinical systems, maximising their chance of being sustained. Thirdly, senior leadership support was seen as critical not just in supporting implementation but also in sustaining longer-term changes brought about by the initiative. Lastly, practitioner incentives were identified as potential levers to engage junior doctors, a crucial part of the acute medical work force and essential to the initiatives, as there is currently little recognition or reward for involvement

**Conclusions:**

The factors identified have been shown to be supportive in the successful implementation of care bundles as a mechanism for implementing clinical guidelines. Addressing these factors at a practitioner and organisational level, alongside the use of a systematic quality improvement approach, should increase the likelihood that care bundles will be implemented successfully to deliver evidence based changes in the acute medical setting.

## Background

Clinical guidelines have been defined as “systematically developed statements to assist practitioner and patient decisions about appropriate health care for specific clinical circumstances” [1]. They are a well-established mechanism for translating research knowledge into guidance to ensure that patients receive care based on the best available evidence by providing recommendations for care processes to reduce unnecessary variations in clinical outcomes [2]. Despite this, consistent implementation of clinical guidelines remains a challenge.

A ‘care bundle’, as defined by the Institute for Healthcare Improvement (IHI), is “a small set of evidence-based interventions for a defined patient segment/population and care setting that, when implemented together, will result in significantly better outcomes than when implemented individually” [3]. The development and implementation of care bundles has been shown to be an effective way of delivering evidence-based care, often based on clinical guidelines, especially within the emergency, acute and critical care settings [3]. Care bundles have been developed for ventilator-acquired pneumonia, myocardial infarction, sepsis and many other conditions [4–6]. Care bundles have also been shown to have some positive impact on clinical outcomes, including reducing mortality [7]. The ‘care bundle’ approach focuses on specific care processes delivered by individual healthcare professionals and provides a flexible method to adapt and contextualise evidence locally within a healthcare organisation; for example, if services are not available or referral processes differ between organisations then bundles can be modified to fit local needs and available resources [8].

The early recognition and treatment of patients with a range of medical conditions requiring urgent care is essential to ensure optimal clinical outcomes. In the UK, as in many other European countries, acute medicine has developed to manage the acutely unwell patients within the first 24–48 h of hospitalisation, but there are challenges to delivering the appropriate, evidence-based care prescribed by clinical guidelines. As such, approaches that increase the likelihood that care bundles (and thus clinical guidelines and evidence-based care) will be successfully implemented in the acute medical setting are particularly needed.

Improving the quality of healthcare, which includes delivering evidence-based care, is complex and numerous barriers exist. Social barriers include engaging the necessary stakeholders, and establishing a common understanding of the problems and potential solutions [9]. Technical barriers exist where the necessary skills and knowledge of implementation strategies and approaches, such as those related to quality improvement (QI), are absent from the clinical team [10].

Whilst QI approaches have long been considered useful to monitor and guide implementation of improvements in care, recently, a greater emphasis has been placed on the supportive social and technical functions of QI tools, such as the Action Effect Method, in the design, planning and implementation of interventions [11]. QI provides a structured approach to bringing together clinicians, researchers, healthcare managers and patients to work to overcome some of these barriers to implementation [12]. Within the National Institute of Health Research (NIHR) Collaboration for Leadership in Applied Health Research and Care (CLAHRC) Northwest London, a range of QI methods, including process mapping, measuring for improvement, Plan-Do-Study-Act cycles, have been adopted to support front line clinical teams to translate research into improvements in healthcare [13]. The programme provided both funding and training for more than 50 different clinical teams across northwest London in the period 2008-13, a region that provides healthcare for more than 2 million people.

### Case studies of the implementation of care bundles

Two implementation initiatives supported by the CLAHRC Northwest London programme were selected for investigation. Each explicitly aimed to implement clinical guidelines through developing a new care bundle for use in the acute medical setting and demonstrated improvements in compliance with process measures. One focused on chronic obstructive pulmonary disease (COPD) and the other on diabetic foot care.

COPD is a long-term respiratory condition that is characterised by breathlessness, frequently resulting in hospitalisation, where mortality and morbidity are often high. Following hospitalisation, consistency in care during admission, discharge and follow-up has been shown to reduce re-admissions and improve clinical outcomes. A COPD care bundle was developed, based on current best practice clinical guidelines, to improve the quality and consistency of the care received by patients, and to reduce variations in care processes and clinical outcomes (COPD Care Bundle—A1) [14].

Diabetic foot complications remain an important cause of morbidity and hospitalisation in the UK, often resulting in lower extremity amputation. Timely identification and management of diabetic foot can prevent significant complications and reduce associated morbidity, improving clinical outcomes. A diabetic foot care bundle was developed to improve screening and management of in-patient diabetic foot complications based on current best practice guidelines (Diabetic Foot Care Bundle—B1) [15, 16]. Table 1 provides a summary of the initiatives, aligned to the IHI definitions of the care bundles [3].

**Table 1:** Overview of implementation initiatives selected as case studies

	COPD Bundle—A1	Diabetic Foot—B1
Duration	04/09- 09/10	04/11- 09/12
Site	Hospital A	Hospital B
Implementation team	Respiratory consultants, clinical nurse specialists, pharmacist, patient advisor, physiotherapist, smoking cessation specialist, project manager (nurse) and QI advisors	Endocrinology consultants, patient advisors, podiatrist ward/specialist nurses, project manager (nurse), research nurse and QI advisors^a^
Resources for implementation	£100,000	£100,000
Evidence-base for bundle	National Institute for Health and Clinical Excellence Clinical guideline 101 (2010) [14]	Diabetes UK Guidelines (2011) and National Institute for Health and Clinical Excellence Clinical guideline 119 (2011) [15, 16]
Defined patient group/segment	Patients admitted to hospital due to an acute exacerbation of COPD	Patients admitted to hospital for any reason with known diabetes
Care setting	Acute medical unit/ward	Acute medical unit/ward
Time to deliver bundle	During admission	During admission
Outcomes to be improved	Reduced readmission to hospital and in hospital mortality	Reduced readmission to hospital and complication of diabetic foot including amputation
Care bundle elements	COPD Bundle for acute exacerbation • Respiratory nurse notified of admission • Smokers offered smoking cessation assistance • Referral to pulmonary rehabilitation for assessment • Provision of written information about COPD • Assessment and demonstration of satisfactory inhaler use • Follow-up appointment with specialist	Diabetic foot bundle • Presence or absence of Ulcers • Presence or absence of infection (Fever, Low BP, Red and/or warm foot, pain without trauma) • Presence or absence of ischaemia (Absent foot pulses, Cold or gangrenous foot) • Presence or absence of deformity [Charcot Foot] (Foot does not look normal) Positive result- manage appropriately
Impact (during initiative)	Care bundle delivered to ≥ 90% of patients	900 patients screened in the first 12 months ^b^
Sustainability (After initiative)	Continued implementation of care bundle	Modified assessment used by surgical team
Diffusion	Care bundle by numerous NHS acute hospital trusts throughout England, supported by CQUIN payments	Clinical lead left the hospital and was continued by surgical lead

^a^The researcher (SG) was also a member of the implementation teams acting as a quality improvement advisor on behalf of the funder (NIHR CLAHRC Northwest London)

^b^Representing approximately 2% of the inpatient population, a likely underrepresentation as current figures suggest inpatient prevalence of 5–30% [25]

This study aimed to identify factors that supported the successful implementation of two care bundles in the acute medical setting that used quality improvement methods.

## Methods

### Data sources

The initiatives underwent 6 and 18-month progress reviews, with one undergoing an additional 12-month review. Prior to the start of the initiatives, the time-points for the reviews were agreed between the implementation teams and CLAHRC Northwest London, who were the funders and provided support with quality improvement methods. In preparation for each review meeting the implementation teams were expected to produce a report with the clinical lead and project manager leading its production but gaining input from the rest of the team. The review itself was attended by the implementation team and chaired by a senior member of CLAHRC Northwest London management team. Activities were intended to be opportunities for each team to reflect on implementation and progress to date. The 18-month reports were based on the Standards for Quality Improvement Reporting Excellence (SQUIRE) guidelines [17]. Contemporaneous data for analysis included the review reports, review minutes and audio recordings of the review meetings, where available, as outlined in Table 2. Verbal consent was sought from participants at the time of the review to audio record the meetings. Recordings were transcribed verbatim by the researcher (SG).

**Table 2:** Overview of data sources available for each case study from progress reviews

Review point	A1-COPD Bundle	B1-Diabetic Foot
6-Month	Review minutes	Review minutes
	Review report	Review report
		Review audio
12-Month	N/A	Review minutes
		Review report
18-Month	Review report	Review minutes
		Review report
		Review audio

### Analytical Approach

Many theories, models and frameworks have been developed to explain and support the implementation of interventions in healthcare, one such example is the Consolidated Framework for Implementation Research (CFIR) [18]. The CFIR, which provides a framework for exploring implementation initiatives, is composed of five domains: *Intervention characteristics, Inner setting, Processes, Characteristics of individuals* and *Outer setting.* Only the first three of the five domains were used as an analytical framework due to constraints imposed by the sources of data, namely that this was a retrospective analysis of data collected during the implementation of the two initiatives.

The domains and sub-domains of the CFIR, as outlined in Table 3, were used to create a coding framework in Nvivo-10 (QSR International, Australia), for a largely deductive analysis of the data, though care was taken to allow for the emergence of themes in the data not contained in the CFIR. The nodes were subsequently exported and used to construct a narrative of the process of implementation, and the sub-domains used to structure the reporting of the analysis and identify factors associated with successful implementation of the care bundles.

**Table 3:** Three of the five domains of the Consolidated Framework for Implementation Research that were used as an analytical framework

Domains	*Intervention characteristics*	*Inner setting*	*Processes*
Sub-domains	Intervention source	Structural characteristics	Planning
	Evidence strength and quality	Networks and communication	Engaging
	Relative advantage	Culture	Executing
	Adaptability	Implementation Climate	Reflecting and evaluating
	Trialability	Readiness for implementation	
	Complexity		
	Design quality and packaging		
	Cost		

*Characteristics of individuals* and *Outer setting* were omitted due to constraints of the data

## Results

### Intervention characteristics

The CFIR outlines the important role that *Intervention characteristics* have been shown to play in determining the success of the implementation of the interventions themselves. The complexity of an intervention can often result in difficulties in implementation and can be characterized by the introduction of non-routine processes or the involvement of multiple steps in the intervention at clinical decision points. The two implementation teams had identified these issues and aimed to ensure that steps in the implementation process were kept to a minimum. Some of the complexity encountered was due to the interface between the intervention and the information systems, and posed problems for the clinical teams. Several of the QI approaches, including the Action Effect Method and process mapping, were recognised by the teams as tools that helped them understand the complexity of the system.

### Inner setting

Analysis of the *implementation climate,* an aspect of the organisational culture that reflects receptiveness to change and new ways of working in the organisation, identified several important issues. These specifically related to one professional group, key to the success of many aspects of the initiatives, namely, junior doctors.

As improvement skills are not recognised as a core skill in UK medical training, there was often little incentive for junior doctors to become involved in these initiatives, although paradoxically the success of the initiative specifically relied on changing their clinical practice.

“It’s mainly that they will have recognition of the work that they are doing, all those junior doctors when they go to their next [rotation]… they have to show that they have been involved in some research project or care pathways. As we support it in your reference we would recognise all the work you have been doing…” [B1]

Additionally, junior doctors were rarely involved in a structured and sustained way, often with no formal recognition as part of the implementation team. Identifying ways to involve junior doctors and provide feedback to support their professional development goals were seen as potential levers for better engagement of these key staff members.

More broadly, this is related to the availability of resources, especially the workforce undertaking the implementation initiatives and the compatibility of the intervention with the needs of those implementing it. As such, members of the implementation teams needed to negotiate with clinical staff affected by the introduction of an intervention to ensure resources were available to implement it effectively. This negotiation was also seen as an opportunity to ‘sell’ the intervention as a development opportunity for the nurses to become involved in the initiative. At an organisational level, aligning the anticipated benefits of the intervention with the needs and priorities of the organisation provided an opportunity to garner support for the initiative.

Acknowledging the needs and pressures of the organisation, and aligning these to the potential benefits of an intervention creates a *tension for change*. This outlines the evidence for changing the status quo*,* and may be supported by external agencies, such as professional bodies, commissioning organisations or patients themselves, which in turn may act as drivers within the organisation.

“Having a patient’s perspective has been tremendously useful in ensuring we provide the best possible patient experience throughout the different stages of the project. Our patients have been integral to the development of the project.” [B1]

The organisational commitment to an initiative is essential not only for the initial success of the implementation, but also for its long-term sustainability. Several factors have been identified that play a significant role in defining organisational commitment, including access to resources, information and knowledge, and the level of support from organisational leaders/executives. Embedding changes in care processes brought about by the implementation of guidelines can be supported through ‘knowledge management’ and data collection systems.

Senior leadership involvement in the initiatives was often minimal with no real lines of accountability between the project managers, usually from outside of the clinical team, and senior clinicians, which occasionally resulted in a conflict of priorities for the initiatives and allocation of resources.

“I think the best way would actually be acknowledgment… they will actually be more motivated to do it. As you say about having that at the end of the reference and all that, ‘cause [sic] they are more into their references… they are very passionate about their portfolios… So, if they get to know that they will have something to take to the next area they are going I think that will motivate them more than anything…” [B1]

The level of engagement from the hospital senior management/executive team across the initiatives was low, despite the contractual agreement between the funder and a named executive sponsor from the host organisation to oversee each initiative. However, other opportunities for engaging with senior leadership were identified and proved particularly useful, such as presenting progress at board meetings. Support from senior leaders/executives was identified as crucial not only for the morale of the implementation team, reinforcing that the work they were doing was deemed important by the organisation as a whole, but also in providing leverage to convince other members of staff to take up the intervention.

“Following consultation with the [Chief Executive Officer]… I will be approaching consultants on an individual basis to raise awareness and the [CEO] has given her support for the project to be highlighted by managers as well as clinicians. Also, following discussion with the [CEO] and [Patient involvement manager], we will be planning to empower patients to request foot examination on admission to hospital.” [B1]

The re-integration of any new process into ‘business as usual’ was identified as being problematic, but made easier with the support of a sponsor from the executive team.

“…each level of management… seeks out the staff implementing the improvements… to support and nurture the staff in this delivery… project delivery teams… are working in isolation of the hosting organisation… where senior staff are involved it is not in a monitoring capacity…nor do they take on the ownership of the project … I am sure it would have a positive impact on sustaining the improvement [A1]

Where implementation teams demonstrated support from the senior leaders/executives, it was shown not only to help explore solutions to some of the problems encountered by the implementation teams but also to encourage some level of ‘ownership’ of the implementation initiative by the executive team.

“We gave an update on the progress last Tuesday in fact, the chairman was there, the non-executive directors and they seemed to be all supportive and were thinking of ways to increase compliance amongst doctors.” [B1]

Examples of ‘knowledge management’ and data collection systems used to support the implementation initiatives included the nursing assessment booklets, a collection of basic nursing tools used by nursing staff to assess patients on admission, and the admission clerking pro-forma, a commonly used tool for systematising the recording of clinical assessments of newly admitted patients by junior doctors. These were frequently separate from the electronic patient record systems (EPR), resulting in duplication of data entry. It appeared that integration of improvements introduced through the implementation initiatives into routine clinical data systems was essential to embed change, thereby maximising the potential for sustainability and conferring a certain level of legitimacy on the new processes of care within the hospital.

“It will spread throughout the whole hospital then…if it’s on the core assessment then it will have to be done automatically”; “This is the first project in [hospital trust] where paperwork has been included in an assessment booklet and this will be seen as beneficial by the Clinical Effectiveness Committee.” [B1]

Resources were also required to provide the necessary skills and training for members of the implementation teams, with further additional dedicated resources to support the initiatives, especially related to project management and data collection/analysis.

“…at that time, we had no research nurse, the doctors were not aware of the project and they were very busy on the acute medical unit… the nursing staff rolled their eyes to heaven as they didn’t appreciate the value of the project as it was an additional assessment in the pack… since we’ve had the improvement nurse and we’ve seen progress the views are much better, the culture has definitely changed. On AAU the nurses now tell me ‘we’ve done these two patients but we’ve haven’t done that one yet’ and I tell them how pleased I am.” [B1]

The provision of supernumerary support in clinical areas also offered a further mechanism for supporting busy clinicians, although this had to be carefully balanced with the risk of developing a dependency on the additional resources, which are often unsustainable, and presents a risk to the longer-term successful implementation.

“…continuing with [the] Diabetic Foot [initiative] without a project manager will be challenging…” [B1]

“…[we need to look] at existing resources within [the hospital] as a way to ensure [the] Diabetic Foot [initiative] carries on successfully. Keeping up a high profile with senior managers is key.” [B1]

In addition to training staff specifically to deliver the initiatives, it was also necessary to engage ward staff in events and workshops in developing the intervention, and provide additional training to develop new skills and improve awareness of the new processes associated with the initiative, both of which required additional staff to cover their clinical duties.

### Process

Planning of implementation initiatives includes the articulation of the underlying process theories to which the implementation of interventions, like care bundles, is contextualised to the specific setting. The range of tools that comprise the comprehensive QI approach supported by CLAHRC Northwest London provided a framework for clinical teams to use for their improvement/implementation initiatives, along with technical QI advisors allocated to each implementation team.

Engaging a range of stakeholders including opinion leaders, ‘champions’ and those with formal implementation roles within the team is recognised as an important aspect of implementation initiatives. This also includes the role of external change agents, who often have the technical knowledge and expertise to support the delivery of initiatives [18]. As already described, one of the biggest challenges identified by the implementation teams was gaining support from the senior/executive team within the organisation, although, when achieved, this often had a significant positive effect on the delivery of the initiatives.

The importance of assembling a multi-professional team and assigning specific roles to members of the team, including a project manager with protected time, was also recognised as important factor. The roles and the amount of time each person could commit to the initiative were outlined in the contractual arrangements, but often these were difficult to enforce due to different lines of accountability among members of the team.

Due to the iterative development of the initiatives, aims that were initially established were constantly reviewed and modified to adapt to changes within the team or organisation. Although there was a sense that the implementation team achieved its objectives and executed its plan, this did not necessarily include ‘embedding’ the intervention to ensure its sustainability.

The training in, and the use of, the QI tools was intended to build capacity in the use of QI approaches. As such, the support offered was seen positively by implementation teams since the skills required to undertake implementation initiatives have not always been seen as core skills for clinical or managerial staff.

## Discussion

Analysis of the two case studies provides an opportunity to examine, in detail, the implementation of care bundles based on clinical guidelines, to identify key factors that directly affect their success.

### Resources for implementation

The implementation teams discussed the importance of dedicated resources for supporting their initiatives, which includes training to develop QI skills within the team, but also the need for capacity building within the organisation. Whilst the provision of specific expertise and technical assistance, especially related to data collection and analysis, was perceived as valuable, the provision of additional clinical staff was also a useful mechanism to engage busy clinicians. On the other hand, the provision of additional resources on which the initiatives might become dependent had to be carefully balanced against longer-term sustainability.

### Perception of the sustainability of change

As part of a strategy to change clinical practice, new care processes needed to be embedded into existing clinical systems to maximise the chance of sustaining change. ‘Knowledge management’ and data collection systems with which practitioners interact, such as nursing assessment booklets, the junior doctor clerking pro-forma and electronic patient record systems (EPRs), offered an opportunity to achieve this. Furthermore, integrating changes within the ‘knowledge management’ and data collection systems conferred legitimacy on the interventions that were part of the initiatives. As a result, it became easier to incorporate these new processes into the routines of the organisation. This ‘normalisation’ of changes within the clinical system aligns with May’s Normalisation Process Theory (NPT) [19]. NPT, an empirically derived theory, brings together the various elements that support or hinder the ‘normalisation’ or ‘routinisation’ of new processes in healthcare.

### Senior leadership support

Senior leadership support was identified as a critical factor not just in ensuring that the implementation initiative was successful but also in sustaining longer-term changes. Despite little support seen through the formal role of executive sponsor, other opportunities for engaging with senior leadership were found. Senior leaders had an important role not only acting as champions for the implementation initiative to encourage staff involvement, but also as brokers between the clinical team and the executive/senior management. Senior leadership engagement was also seen as important in ensuring integration of the new process into ‘business as usual’. The importance of this was further underlined by the need for alignment between initiatives and organisational priorities. Demonstrating how the initiative directly or indirectly linked to wider organisational needs was helpful in garnering support from senior leaders.

### Practitioner incentives

Despite the recognition of the importance of QI skills by organisations that oversee the training of doctors, including the UK General Medical Council and Academy of Medical Royal Colleges, this has not yet become part of post-graduate medical education [20]. Current QI involvement of junior doctors, who are a crucial part of the medical work force in the acute medical setting, is based on good-will. Structured processes for their involvement and appropriate feedback mechanisms may be beneficial in improving their engagement. Furthermore, harnessing the NHS’ Commissioning for Quality and Innovation (CQUIN) programme, a system that links hospital payments by commissioners to the demonstrable delivery of prescribed evidence-based care processes, could offer a further lever for such initiatives [21, 22]. Whilst this mechanism is not necessarily a direct incentive at the practitioner or clinical team level, it does offer the potential to provide extra financial resources that could be linked to departments, specialties or teams. This may be analogous to the Quality and Outcomes Framework (QOF) in NHS general practice, which provides practice level financial incentives for attaining quality targets [22]. This may also provide a further opportunity to align the monitoring of care processes with implementation of clinical guidelines, where care bundles are developed from specific clinical guidelines that are subsequently linked to performance incentives [23]. However, there is a risk that this could promote ‘gaming’, which may happen when performance indicators are linked to payment [24].

### Strengths and limitations of this study

The implementation initiatives were chosen as they demonstrated the successful implementation of clinical guidelines using care bundles in the acute medical with methodological consistency with regards to the use of QI methods. This does however limit the generalisability of the findings, especially when applied to implementation teams using a different QI approach or different assessments of success criteria. However, demonstrating the success of these initiatives in the acute medical setting, which provides a challenging environment, could indicate likely success in less constrained settings. A further methodological limitation lies in the data sources, which whilst generated contemporaneously, do limit their content.

## Conclusion

Developing an evidence-based approach to implementation, and not just leaving this to chance, should be seen as important as the research that underpins the evidence in the clinical guidelines that often forms the basis of the care bundles themselves. Moreover, delineating the processes that supports the implementation of care bundles provides opportunities to better plan and manage implementation strategies. The factors identified in the study have been shown to support implementation of care bundles and addressing these at the practitioner and organisational level, alongside the use of a systematic quality improvement approach, should increase the likelihood of successful implementation of care bundles in the acute medical setting.

## Abbreviations

CEO: Chief Executive Officer
CFIR: Consolidated Framework for Implementation Research
CLAHRC: Collaboration for Leadership in Applied Health Research and Care
COPD: Chronic obstructive pulmonary disease
CQUIN: Commissioning for Quality and Innovation
EPRs: electronic patient record systems
NICE: National Institute for Health and Care Excellence
NIHR: National Institute of Health Research
NPT: Normalisation Process Theory
QI: Quality improvement
QOF: Quality and Outcomes Framework
SQUIRE: Standards for Quality Improvement Reporting Excellence
